# Interfacial Engineering of Sustainable Microcrystalline Cellulose-Reinforced PLA/PHA Biocomposites for Enhanced Performance

**DOI:** 10.3390/polym18151895

**Published:** 2026-08-01

**Authors:** Usman Saeed

**Affiliations:** Department of Chemical and Materials Engineering, Faculty of Engineering, King Abdulaziz University, Jeddah 21441, Saudi Arabia; umsaeed@kau.edu.sa

**Keywords:** polylactic acid (PLA), polyhydroxyalkanoate (PHA), microcrystalline cellulose (MCC), biocompatibility, biodegradability

## Abstract

The increasing demand for sustainable materials has accelerated the development of biodegradable polymer composites with enhanced multifunctional performance for engineering, packaging, and biomedical applications. In this study, poly(lactic acid) (PLA)/polyhydroxyalkanoate (PHA) biocomposites reinforced with microcrystalline cellulose (MCC) and a compatibilizer were fabricated by melt blending followed by compression molding. Fourier-transform infrared spectroscopy confirmed enhanced hydrogen-bonding interactions between MCC and the PLA/PHA matrix, indicating improved interfacial compatibility. X-ray diffraction and Differential scanning calorimetry revealed that MCC acted as an significant heterogeneous nucleating agent, increasing the crystallinity from 28.6% for the neat PLA/PHA blend (S0) to 40.1% while reducing the cold crystallization temperature from 115.2 to 110.5 °C and increasing the melting enthalpy from 29.8 to 38.9 J g^−1^. Thermogravimetric analysis demonstrated improved thermal stability, with the maximum degradation temperature increasing from 325.1 to 343.8 °C and the residual char yield increasing from 5.6% to 16.8%. Specimen S7, containing 6 wt.% MCC and 2 wt.% compatibilizer exhibited the optimum overall performance, achieving a tensile strength of 64 MPa, Young’s modulus of 2500 MPa, impact strength of 5.8 kJ/m^2^, cell viability of 98%, and 88.5% weight loss after 180 days of soil burial. These findings demonstrate that interfacial engineering with MCC and compatibilizer significantly enhances the structural, thermal, mechanical, biological, and biodegradation performance of PLA/PHA biocomposites, making them promising candidates for sustainable advanced packaging and biomedical applications.

## 1. Introduction

The widespread use of petroleum-based plastics and the resulting environmental challenges have intensified global efforts to develop sustainable, biodegradable materials with reduced ecological impact. Among these, bio-based polymer composites have emerged as promising candidates for packaging, agricultural, and engineering applications owing to their renewability, biodegradability, and favorable performance characteristics [1,2,3]. Conventional plastics possess excellent mechanical performance and durability; however, their resistance to degradation has resulted in severe environmental pollution and increasing collection of plastic waste in terrestrial and oceanic ecosystems [4]. Consequently, significant research efforts have been directed toward the development of bio-based and biodegradable polymeric materials that can reduce dependence on fossil resources while minimizing environmental impacts. Among the wide range of bio-based polymers, poly(lactic acid) (PLA) and polyhydroxyalkanoates (PHA) have been widely explored as environmentally benign materials owing to their renewable origin, biodegradability, biocompatibility, and favorable processing characteristics [5,6,7]. PLA is one of the most widely commercialized biodegradable thermoplastics due to its transparency, high stiffness and ease of processing using conventional polymer manufacturing techniques. Nevertheless, its natural brittleness, relatively low thermal stability and limited toughness restrict its application in demanding structural and packaging environments [8,9,10]. PHA, on the other hand, exhibits excellent biodegradability and superior ductility compared with PLA, making it a fascinating candidate for improving the mechanical performance of PLA-based materials. Blending PLA with PHA has therefore become an effective strategy to achieve a balanced combination of stiffness, toughness, and biodegradation behavior [11,12]. However, phase incompatibility and weak interfacial interactions between the two polymers may limit the overall performance of PLA/PHA blends, necessitating the incorporation of suitable reinforcement materials and compatibilization approaches. Natural cellulosic fillers have received considerable attention as sustainable strengthening for biodegradable polymer matrices due to their low density, high specific strength, biodegradability, renewability, and widespread availability [13,14,15,16]. Among these fillers, microcrystalline cellulose (MCC) is particularly attractive because of its high crystallinity, large specific surface area, excellent mechanical properties, and abundant hydroxyl functional groups [17,18,19]. The integration of MCC into biodegradable polymer matrices can improve stiffness, dimensional stability, thermal resistance, and crystallization behavior while maintaining the environmentally friendly characteristics of the composites. Furthermore, MCC can act as an efficient heterogeneous nucleating agent, promoting crystal formation within polymer matrices and enhancing overall material performance [20,21,22]. Despite these advantages, the hydrophilicity of MCC and the relatively hydrophobic characteristics of PLA and PHA often result in inadequate interfacial compatibility, filler agglomeration, and inefficient stress transfer between the polymer matrix and reinforced fiber. These limitations may reduce the reinforcing efficiency of MCC and negatively affect the mechanical and thermal properties of the resulting composites [23,24,25]. To overcome these challenges, compatibilizers are commonly employed to improve filler dispersion, strengthen interfacial adhesion, and enhance the overall structural integrity of the composite system. Improved interfacial interactions facilitate stress transfer across the matrix–reinforcement interface and promote more homogeneous microstructures, leading to superior performance [26,27]. Recent studies have demonstrated that cellulose-reinforced PLA-based composites exhibit enhanced mechanical properties, thermal stability, and crystallinity compared with unfilled polymers. Similarly, the addition of compatibilizers has been shown to improve filler dispersion and reduce interfacial defects. However, the combined effects of MCC concentration and compatibilizer content on the thermal, structural and morphological characteristics of PLA/PHA biocomposites remain insufficiently understood [28,29,30]. In particular, identifying the optimum balance between MCC loading and compatibilizer concentration is essential for maximizing crystallinity, thermal resistance, and microstructural homogeneity while avoiding filler agglomeration and deterioration of composite performance [31,32,33].

The present study aims to fabricate and evaluate PLA/PHA/MCC biocomposites containing varying MCC concentrations and compatibilizer contents. A systematic investigation was conducted to assess the influence of MCC and compatibilization on the structural, thermal, and morphological characteristics of the composites. Fourier-transform infrared spectroscopy (FTIR) was employed to examine intermolecular interactions and chemical compatibility, while X-ray diffraction (XRD) analysis was used to assess crystalline phase development and crystallinity evolution. Thermogravimetric analysis (TGA) and derivative thermogravimetry (DTG) were performed to evaluate thermal stability and degradation behavior. In addition, scanning electron microscopy (SEM) was utilized to investigate filler dispersion and interfacial morphology. The novelty of this work lies in the comprehensive correlation of crystallinity, thermal stability, and microstructural characteristics with varying MCC and compatibilizer concentrations in PLA/PHA-based biocomposites. The findings provide valuable insights into the role of MCC as a nucleating and reinforcing agent and highlight the significance of compatibilization in achieving optimal matrix–filler interactions. The developed biocomposites are expected to offer improved thermal performance, enhanced structural integrity, and superior sustainability, making them promising candidates for environmentally friendly packaging, consumer products, and lightweight engineering applications.

## 2. Materials and Methods

### 2.1. Materials

Polylactic acid (PLA 2003D) pellets (NatureWorks, Plymouth, MN, USA) having melt flow index of 6 g/10 min and polyhydroxyalkanoate (PHA) pellets (NatureWorks LLC, Plymouth, MN, USA) with melt flow index of 5.76 g/10 min were selected as the biodegradable polymer matrix. Microcrystalline cellulose (MCC, Type 101) having particle size of 5 μm procured from Vivion Inc. (Gardena, CA, USA), was incorporated as a bio-derived reinforcing agent. To improve the interfacial interaction between the hydrophobic PLA/PHA matrix and the hydrophilic MCC, malaeic anhydride grafted compatibilizer, Fusabond (MB100D DuPont, Wilmington, DE, USA), was incorporated promoting stronger interfacial adhesion, improved filler dispersion, and more efficient stress transfer in the resulting biocomposites.

### 2.2. Preparation

PLA, PHA, MCC, and the compatibilizer were placed in a vacuum oven at 60 °C for 24 h prior to processing forresidual moisture removal. The required amounts of PLA, PHA, MCC, and compatibilizer were then premixed according to the formulations listed in Table 1. The mixing blends were melt compounded using a twin screw extruder at a temperature range of 170–190 °C and with a screw speed of 60 rpm to ensure uniform dispersion of MCC within the PLA/PHA matrix. The extruded strands were cooled at room temperature, pelletized, and subsequently compression molded into sheets using a hot press at 170 °C under 10 MPa pressure for 5 min followed by cooling to room temperature. The molded sheets were cut into the required dimensions for thermal, mechanical, morphological, biodegradation, and biological characterization.

### 2.3. Characterization

The surface morphology was characterized using a scanning electron microscope (JEOL, Tokyo, Japan—JSM-7600F) operated at an accelerating voltage of 15 kV, and the micrographs were recorded at a magnification of 5000×. Prior to SEM imaging, specimens were deposited with thin conductive gold layer to improve electrical conductivity and suppress surface charging during imaging.

Fourier Transform Infrared Spectroscopy (FTIR, Thermo Fisher Scientific, Waltham, MA, USA) was employed to determine the chemical structure and functional groups of the PLA/PHA/MCC biocomposites. Infrared spectra were acquired between 4000 and 400 cm^−1^ in attenuated total reflectance (ATR) mode.

The crystalline characteristics of the PLA/PHA/MCC biocomposites were evaluated utilizing an X-ray diffractometer (XRD-7000, Shimadzu Corporation, Tokyo, Japan). Diffraction measurements were performed with Cu Kα radiation having wavelength λ of 1.5406 Å at an voltage of 40 kV and a tube current of 30 mA. The diffraction patterns were collected over a 2θ range of 5–50° using a scanning speed of 2° min^−1^ with a step interval of 0.02°. The XRD patterns were analyzed using OriginPro 2024 software. Prior to peak analysis, background correction was performed to eliminate instrumental noise and scattering effects. The diffraction profiles were subsequently deconvoluted using Gaussian–Lorentzian peak fitting to separate the crystalline reflections from the broad amorphous halo. The integrated areas corresponding to the crystalline peaks (Ac) and amorphous region (Aa) were determined after peak fitting, and the degree of crystallinity (Xc) was calculated by using the following Equation (1):

(1) $$X_{C}\left(\%\right)=\frac{A_{C}}{A_{C}+Aa}\times 100$$

where Xc is degree of crystallinity (%), A_c_ presents the total integrated area of the crystalline peaks, and A_a_ denotes the integrated region of the amorphous region.

The crystallization behavior and thermal transitions of the PLA/PHA/MCC biocomposites were investigated using Differential Scanning Calorimetry (DSC 200 F3, Netzsch, Selb, Germany). Approximately 10 mg of each biocompositespecimen was encapsulated in a sealed aluminum pan and analyzed under a nitrogen environment. The thermal cycle comprised heating from 25 °C to 220 °C at a rate of 10 °C min^−1^ followed by cooling to room temperature. The degree of crystallinity (Xc) was evaluated by following Equation (2):

(2) $$X_{C}\%=\frac{{∆H}_{m}-{∆H}_{CC}}{{∆H}_{m}^{o}\times w}\times 100$$

where ΔH_m_ is the melting enthalpy of the sample, ΔH_cc_ is the cold crystallization enthalpy, w is the weight fraction of PLA in the composite and ${∆H}_{m}^{o}$ the enthalpy of fusion of 100% crystalline PLA (93.7 J g^−1^).

Thermogravimetric analysis (TGA, Setaram, Francheville, France) was employed to examine the thermal stability of the PLA/PHA/MCC biocomposites. Thermogravimetric measurements were performed using 10 mg of each biocomposite specimen placed in alumina crucibles under a nitrogen purge. The specimens were heated from room temperature to 800 °C at a constant heating rate of 10 °C min^−1^ and the corresponding mass-loss profiles were recorded to evaluate their thermal stability and degradation behavior.

Tensile testing was performed using a universal testing machine (3369 Instron, Norwood, MA, USA) according to ASTM D638 [34]. Dog-bone-shaped specimens were tested at 25 °C with preload of 0.50 N and crosshead speed of 5 mm min^−1^. Charpy impact strength was evaluated using a impact tester (JB W300J, Test lab, Warsaw, Poland) in accordance with ASTM D6110 [35]. The specimens were prepared with dimensions of approximately 10 mm × 10 mm × 55 mm prior to testing. At least three specimens were tested for each formulation, and the average values were reported.

The in vitro biological performance of the developed PLA/PHA/MCC biocomposites was assessed through Live/Dead fluorescence staining, the MTT cell viability assay. Before biological evaluation, all specimens were sterilized by immersion in 70% ethanol, thoroughly rinsed with sterile phosphate-buffered saline (PBS), and subsequently subjected to ultraviolet (UV) irradiation for 30 min. Cells were cultured on the sterilized specimen surfaces under standard cell culture conditions using Dulbecco’s Modified Eagle Medium (DMEM) supplemented with 10% fetal bovine serum (FBS) and 1% penicillin–streptomycin. Following the designated incubation period, cell morphology and viability were qualitatively examined using a Live/Dead Cell Staining Kit containing calcein-AM and propidium iodide (PI). Fluorescence images were acquired using an inverted fluorescence microscope (Thermo Fisher Scientific, Waltham, MA, USA) to evaluate cell attachment, spreading, and viability on the composite surfaces. Cell viability was quantitatively evaluated using the MTT assay. After incubation with the MTT reagent, the generated formazan crystals were dissolved in dimethyl sulfoxide (DMSO), and the absorbance was measured at 570 nm using a MultiskanSkyHigh microplate spectrophotometer (Thermo Fisher Scientific, Waltham, MA, USA). Cell viability was expressed relative to the control group. Cell viability was calculated by comparing the absorbance of the biocomposite specimens with that of the untreated control group and expressed as a percentage, shown in Equation (3):

(3) $$CellViability,\%=\frac{A_{s}}{A_{C}}\times 100$$

where As is the absorbance of the sample and Ac is the absorbance of the control.

Finally, the biodegradation behavior of the PLA/PHA/MCC biocomposites was evaluated using a soil burial test. Square specimens of 30 mm × 30 mm × 2 mm) with an average initial weight of 1.20 ± 0.05 gm were embedded in untreated natural soil at a depth of approximately 5 cm to evaluate their biodegradation behavior. At predetermined intervals of 15, 30, 45, 60, 75, 90, 120, 150, and 180 days specimens were carefully retrieved, rinsed with distilled water to eliminate residual soil particles, and oven-dried at 50 °C until a constant mass was attained. Following the drying step, the mass of each specimen was measured using a calibrated analytical balance with a precision of ±0.1 mg. The percentage weight loss was evaluated according to Equation (4):

(4) $$WeightLoss,\%=\frac{W_{0}-W_{t}}{W_{0}}\times 100$$

where W_0_ is the initial weight and W_t_ is the weight after burial for a specified duration. Surface degradation and physical disintegration of the specimens were further monitored through visual observations and optical imaging during the biodegradation process.

The surface morphology and visual degradation behavior of the PLA/PHA/MCC biocompositespecimens during the soil burial test were examined using optical microscope (Leica DM4000B, Leica, Wetzlar, Germany) and the mages were captured at a magnification of 10×.

## 3. Results and Discussion

**Figure 1:**
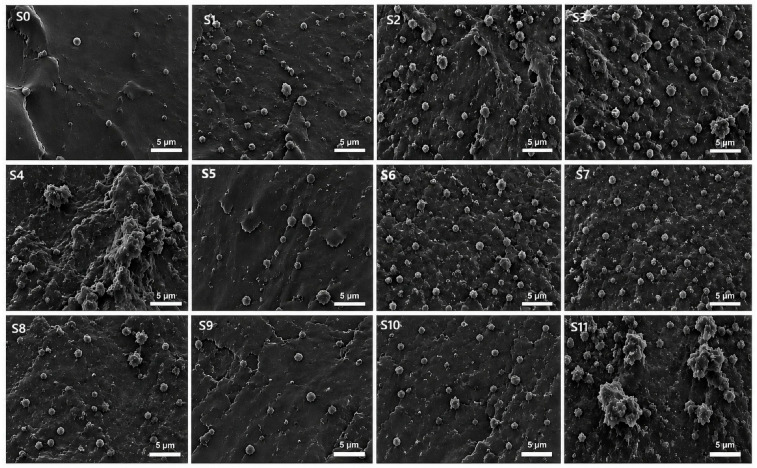
SEM micrographs of the surface morphology of PLA/PHA/MCC biocomposites.

The SEM micrographs presented in Figure 1 illustrate the surface morphology of PLA/PHA/MCC biocomposites containing different MCC loadings and compatibilizer concentrations. The neat PLA/PHA blend (S0) exhibits a relatively smooth and homogeneous surface with few visible irregularities, indicating a continuous polymer matrix and the absence of reinforcing fillers. The surface appears dense and compact, reflecting the typical morphology of an unfilled polymer blend. Although the commercially supplied MCC possessed an average particle size of approximately 50 μm, the filler appeared considerably 2–3 μm in the SEM micrographs. This is attributed to partial fragmentation of MCC during twin-screw melt compounding under high shear conditions, together with improved particle dispersion promoted by the compatibilizer. Furthermore, the SEM images primarily reveal fractured particle sections embedded within the polymer matrix rather than the original particle dimensions. With the incorporation of MCC at low concentrations (S1 and S2), numerous spherical and irregular particulate features become visible on the composite surface. These particles are relatively well dispersed throughout the matrix, suggesting adequate filler distribution during processing. The increase in surface roughness compared with S0 corroborates the successful integration of MCC within the PLA/PHA matrix. At these concentrations, the particles remain largely separated from one another, indicating minimal agglomeration and reasonably good matrix–filler interaction. Further increasing the MCC content to 6 wt.% (S3) results in a consistent distribution of cellulose particles across the surface. The filler particles appear finely dispersed, and the matrix maintains a relatively continuous morphology without extensive clustering. This observation suggests that the MCC particles act as effective nucleating sites while remaining sufficiently separated to maximize their reinforcing potential [36]. At 8 wt.% MCC (S4), the surface becomes noticeably rougher, and localized particle-rich regions begin to appear. Although the overall dispersion remains acceptable, the higher filler concentration increases the probability of particle-particle interactions, which can promote the formation of small agglomerates. Such localized accumulations may partially restrict polymer chain mobility and contribute to the slight reduction in crystallinity observed at higher MCC contents [37]. The effect of maleic anhydride is particularly evident in samples S5 to S8. Compared with the non-compatibilized specimens, these composites exhibit a more uniform particle distribution and improved surface homogeneity. The malaeic anhydride compatibilizer enhances the interfacial interaction between the hydrophilic MCC particles and the hydrophobic PLA/PHA matrix reducing particle aggregation and promoting better wetting of the filler surface by the polymer. Among all formulations, S7 displays the most homogeneous morphology, characterized by uniformly distributed MCC particles and a relatively smooth continuous matrix. The absence of large agglomerates indicates efficient interfacial adhesion and improved dispersion quality. A slight deterioration in surface uniformity is observed for S8, which contains the highest MCC concentration among the compatibilized composites [38]. Small clusters and localized filler-rich regions become more apparent, suggesting that excessive MCC loading may exceed the dispersion capability of the matrix. Although compatibilization still improves filler distribution compared with the non-compatibilized counterpart, some degree of agglomeration is unavoidable at elevated filler concentrations. The highly compatibilized formulations from S9 to S11 exhibit improved surface coverage and enhanced matrix–filler integration. Samples S9 and S10 maintain relatively uniform particle dispersion, indicating that the increased compatibilizer concentration contributes to improved interfacial bonding. However, S11 displays the appearance of larger particle aggregates and irregular protrusions on the surface. These features suggest that excessive MCC content, even in the presence of higher compatibilizer levels, can promote filler-filler interactions and agglomeration. The formation of such aggregates may reduce the effective surface area available for stress transfer and limit further improvements in crystallinity and thermal performance. The SEM observations demonstrate that MCC incorporation significantly modifies the surface morphology of PLA/PHA composites by increasing surface roughness and introducing well-defined particulate features. The malaeic anhydride compatibilizer plays a vital role in enhancing MCC dispersion and interfacial compatibility, resulting in a more homogeneous microstructure. These results confirm that an optimal combination of MCC loading and malaeic anhydride compatibilizer concentration is essential for achieving a well-dispersed microstructure and maximizing the performance of PLA/PHA/MCC biocomposites.

**Figure 2:**
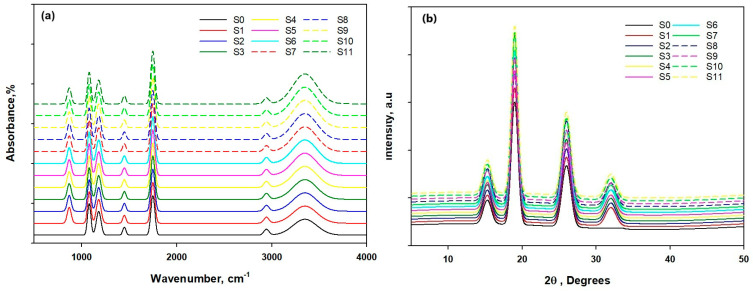
(**a**) FTIR spectra. (**b**) XRD patterns of PLA/PHA biocomposites.

Figure 2a presents the FTIR spectra of the PLA/PHA/MCC biocomposites containing different microcrystalline cellulose (MCC) and malaeic anhydride compatibilizer contents (S0–S11). The spectra of all formulations exhibit the characteristic absorption bands of PLA and PHA indicating that the fundamental chemical structures of both polymers remain intact after melt compounding and compression molding. A broad band centered between 3250 and 3500 cm^−1^ is accredited to the stretching vibration of hydroxyl (O–H) groups originating from the cellulose structure and adsorbed moisture [38]. The intensity of this band progressively increases from S0 to S11 due to the increasing MCC content. Furthermore, the slight broadening of the O–H stretching band indicates the establishment of intermolecular hydrogen bonds involving the hydroxyl groups of MCC and the carbonyl groups of PLA and PHA. These hydrogen-bonding interactions strengthen the interfacial interactions between the hydrophilic cellulose filler and the relatively hydrophobic polymer matrix. The strong absorption peak observed at approximately 1750–1760 cm^−1^ relates to the ester carbonyl (C=O), which is the characteristic functional group of both PLA and PHA. Although no significant shift in the carbonyl peak position is observed, a slight reduction in peak intensity together with minor broadening can be detected in the compatibilized composites from S5 to S11. This behavior suggests that the carbonyl groups participate in secondary intermolecular interactions rather than undergoing any chemical transformation, confirming that melt processing does not alter the polymer backbone [39]. The absorption bands located at 1450 cm^−1^ are correlated to the asymmetric and symmetric deformation vibrations of –CH_3_ groups, while the peak near 1360 cm^−1^ is connected with C–H bending vibrations of PLA and PHA. These peaks remain nearly unchanged for all formulations, demonstrating that the aliphatic backbone of the biodegradable polymers is preserved during processing. The absorption band around 1180 cm^−1^ corresponds to the C–O–C stretching vibration PLA/PHA and presents the ester linkage together with the C–O stretching vibration of cellulose. As the MCC concentration increases, these bands become progressively more intense, reflecting the higher concentration of glycosidic ether linkages contributed by cellulose. The enhancement of this region also supports the homogeneous incorporation of MCC within the polymer matrix. A distinct absorption band observed near 1080 cm^−1^ is attribute to the C–O stretching vibration of cellulose. The gradual increase in its intensity from S0 to S11 validates the successful incorporation of MCC into the PLA/PHA matrix. Likewise, the absorption band at 870 cm^−1^ presents the β-glycosidic linkage of cellulose which becomes more pronounced with increasing MCC loading. The unchanged FTIR spectra, characterized by the absence of new absorption bands after melt blending, indicate that the composite fabrication process was governed primarily by physical interactions, such as hydrogen bonding, rather than the formation of new covalent bonds. Among all formulations, S7 having 6 wt.% MCC with 2 wt.% malaeic anhydride compatibilizer exhibits the most balanced FTIR profile, characterized by a relatively strong O–H stretching band and enhanced C–O–C absorption without excessive broadening or distortion of the ester carbonyl peak. This observation suggests the optimum interfacial interaction between MCC and the PLA/PHA matrix. At higher MCC loadings (S10 and S11), the further increase in hydroxyl absorption intensity reflects the larger cellulose content; however, excessive filler may promote particle agglomeration, reducing the effectiveness of stress transfer despite the stronger O–H signal.

Figure 2b presents the XRD patterns of PLA/PHA/MCC biocomposites. The diffraction profiles reveal the coexistence of crystalline phases originating from the PLA/PHA matrix and microcrystalline cellulose (MCC), confirming the successful incorporation of cellulose into the polymer blend. The neat PLA/PHA blend (S0) exhibits characteristic diffraction peaks at approximately 16.72°, 19.10°, and 25.32°, analogous to the (110), (203), and (200) crystallographic planes of the α-crystalline phase of PLA/PHA. These peaks indicate the semi-crystalline nature of the PLA/PHA polymer. The crystallinity index (Xc) of S0 was calculated as 28.6%, reflecting the relatively limited crystal development in the absence of nucleating fillers. Upon the addition of MCC, diffraction peaks emerge at approximately 32.1° and 25.32° analogous to the (110) and (200) planes of cellulose I.The diffraction peak observed at approximately 25.32°, corresponds to the (200) crystallographic plane of cellulose I and represents the characteristic crystalline reflection of microcrystalline cellulose (MCC). The diffraction feature located near 25.3° arises from the overlapping crystalline reflections of the PLA/PHA matrix and cellulose I. These diffraction peaks are partially superimposed, peak deconvolution was performed following baseline correction to distinguish the crystalline contribution from the amorphous background before calculating the degree of crystallinity. With increasing MCC loading, the intensity of this peak gradually increased, confirming the successful incorporation of cellulose into the PLA/PHA matrix. The increase in peak intensity confirms the successful incorporation of MCC into the polymer matrix. The crystallinity increased to 30.2%, suggesting that cellulose particles acted as heterogeneous nucleation sites that promoted crystal formation during cooling. Further increasing the MCC content to 4 wt.% (S2) produced a noticeable enhancement in diffraction intensity, especially near 16.8° and 25.32°. The increase in crystallinity to 32.8% indicates that additional cellulose particles provided a greater number of nucleation sites, thereby facilitating crystal growth and improving molecular packing within the PLA/PHA matrix. For S3 (6 wt.% MCC), the characteristic diffraction peaks became well defined, with a significant increase in the intensity of the 25.32° (200) reflection. This behavior demonstrates the strong nucleating effect of MCC and its ability to promote ordered crystal formation. Consequently, the crystallinity increased to 34.9%, reflecting improved structural organization. Increasing the MCC loading to 8 wt.% (S4) resulted in a further increase in diffraction intensity, with the crystallinity reaching 36.5%. Although the cellulose content continued to enhance crystal formation, slight peak broadening suggests the onset of localized particle agglomeration, which may limit further improvements in crystal perfection. The incorporation of 2 wt.% malaeic anhydride compatibilizer in formulations from S5 to S8 substantially modified the diffraction behavior. Compared with their corresponding non-compatibilized counterparts, these composites exhibited sharper and narrower diffraction peaks with greater intensity, indicating improved dispersion of MCC and stronger matrix–filler interactions. The compatibilizer enhanced the interfacial adhesion between the hydrophilic cellulose and the hydrophobic PLA/PHA matrix, allowing cellulose particles to act as more efficient nucleating agents. The highest crystallinity of 40.7% was observed for S7 and is attributed to the synergistic effect of 6 wt.% MCC and 2 wt.% compatibilizer. MCC acts as a heterogeneous nucleating agent, promoting crystal nucleation, while the compatibilizer improves matrix–filler interfacial adhesion and filler dispersion, facilitating more uniform crystal growth. At higher filler or compatibilizer contents (S8–S11), particle agglomeration and restricted polymer chain mobility reduce crystal perfection, leading to a slight decrease in crystallinity. The superior crystallinity indicates that the combination of moderate MCC loading and compatibilizer provides optimum filler dispersion and promotes efficient crystal nucleation without significant particle aggregation. These findings are consistent with the DSC results, which showed increased melting enthalpy and reduced cold crystallization temperature, as well as the enhanced mechanical and thermal properties observed for S7. When the compatibilizer concentration was increased to 4 wt.% (S9–S11), the diffraction peaks remained relatively intense but became slightly broader compared with S7. Although crystallinity remained higher than that of the neat blend, a slight reduction was observed (38.3–36.8%). This decrease is attributed to partial MCC agglomeration and excessive compatibilizer which may restrict polymer chain mobility and reduce the efficiency of crystal growth [40]. Nevertheless, the diffraction peaks remained sharper than those of S0 indicating that the overall crystalline structure was still enhanced compared with the unreinforced blend.

**Figure 3:**
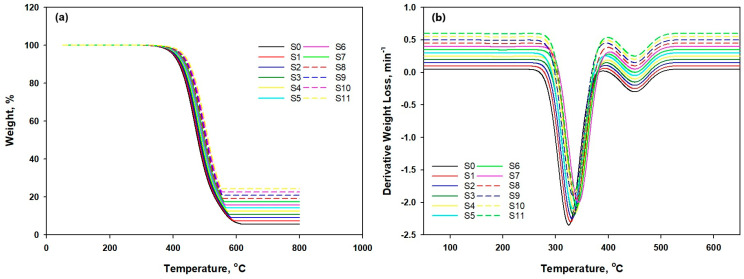
(**a**) Thermogravimetric analysis (TGA) curves showing weight loss with increasing temperature. (**b**) Derivative thermogravimetric (DTG) curves illustrating the corresponding degradation rate.

Figure 3 illustrates the TG and DTG curves of PLA/PHA/MCC biocomposites (S0–S11) containing different MCC loadings and compatibilizer concentrations. As illustrated in Figure 3a, all formulations exhibit a single dominant degradation stage between approximately 280 and 400 °C, indicating that the thermal decomposition of the PLA/PHA matrix occurs predominantly through one major degradation process. The absence of additional degradation steps suggests good compatibility between the polymer matrix and the incorporated MCC, although slight variations in the degradation profile are observed with increasing filler and compatibilizer contents. Also, the corresponding thermal degradation parameters are summarized in Table 2. The neat PLA/PHA blend (S0) exhibits the lowest thermal stability with a Tonset of 301.2 °C and Tmax of 325.1 °C, indicating the relatively early degradation of the polymer matrix. Incorporation of MCC progressively shifts both Tonset and Tmax toward higher temperatures. For thenon-compatibilized composites (S1–S4), Tonset increases from 306.8 to 319.2 °C, while Tmax rises from 328.7 to 336.8 °C. This enhancement demonstrates that MCC acts as an effective reinforcing phase capable of delaying thermal decomposition by restricting polymer chain mobility and promoting the formation of thermally stable char during pyrolysis. The compatibilized formulations (S5–S8) exhibit consistently higher degradation temperatures than their corresponding non-compatibilized counterparts, indicating improved matrix–filler interfacial adhesion and more homogeneous MCC dispersion. Among all formulations, S7 (6 wt.% MCC + 2 wt.% compatibilizer) exhibits the highest thermal stability with Tonset of 323.6 °C and Tmax of 343.8 °C. The enhanced thermal resistance of S7 is attributed to the synergistic effect of well-dispersed MCC particles and improved interfacial interactions, which effectively retard heat transfer and suppress the evolution of volatile degradation products during thermal decomposition. The residual char yield also increases significantly with increasing MCC and compatibilizer contents. The neat PLA/PHA blend produces only 5.6 wt.% residue at 800 °C, whereas the residue gradually increases to 11.8 wt.% for S4. The compatibilized composites exhibit even higher char yields, reaching 16.8 wt.% for S7 and 24.9 wt.% for S11. The higher residual mass originates from the carbonaceous structures generated during pyrolysis together with the thermally stable cellulose-derived char, which acts as a protective barrier against further thermal degradation. Although S11 exhibit the highest char residues, their onset degradation temperatures are lower than that of S7, suggesting that excessive MCC and compatibilizer contents promote greater char formation but do not necessarily provide the optimum overall thermal stability. This behavior is likely associated with localized filler agglomeration and restricted polymer chain packing at higher reinforcement levels.

The DTG curves, Figure 3b, provide additional insight into the degradation kinetics. The primary DTG peak appearing between 325 and 344 °C corresponds to the decomposition of PLA and PHA through ester bond cleavage and chain scission reactions. Such behavior is typical of neat polyester systems, where thermal degradation proceeds mainly through random ester bond cleavage and depolymerization reactions The gradual shift of Tmax from 325.1 °C (S0) to 343.8 °C (S7) confirms the effectiveness of MCC and compatibilizer in delaying degradation. Furthermore, the DTG peak intensity decreases and broadens with increasing MCC content, indicating a reduction in degradation rate and a more gradual decomposition process. At higher temperatures, the remaining carbonaceous structures undergo further thermal decomposition, resulting in the secondary DTG shoulder and the final residual char. A secondary DTG shoulder is observed between approximately 420 and 470 °C, particularly for MCC-containing composites. This secondary degradation event is attributed to cellulose decomposition, char rearrangement, and oxidation of residual carbonaceous structures. The prominence of this secondary peak increases with MCC concentration, confirming the contribution of cellulose to char formation and thermal stabilization. The TG and DTG analyses demonstrate that MCC effectively enhances the thermal stability of PLA/PHA composites, while compatibilizer addition further improves matrix–filler interactions and thermal resistance. The S7 formulation (6 wt.% MCC + compatibilizer) exhibited the highest Tmax (343.8 °C) and the most balanced thermal performance, indicating optimal filler dispersion and interfacial adhesion.

**Figure 4:**
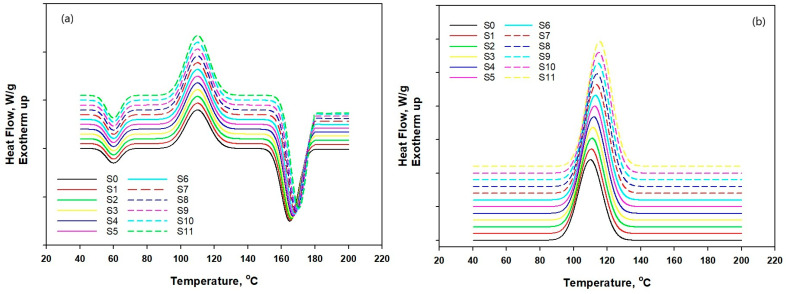
DSC thermograms of PLA/PHA/MCC biocomposites with varying microcrystalline cellulose (MCC) and compatibilizer contents. (**a**) Heating. (**b**) Cooling.

Figure 4 demonstrates the DSC thermograms of the PLA/PHA/MCC biocomposites, and the corresponding second heating cycle thermal parameters are presented in Table 3. The DSC thermograms exhibited the melting temperature (Tm), cold crystallization (Tcc) and glass transition (Tg) reflecting the molecular mobility and crystallization behavior of the biocomposites. The neat PLA/PHA blend (S0) exhibited a glass transition temperature (Tg) of 60.1 °C, a cold crystallization peak at 115.2 °C, and a melting temperature of 168.4 °C, with a degree of crystallinity (Xc) of 28.6%. The relatively high Tcc value indicates that a extensive amount of thermal energy is required to induce crystallization during heating, suggesting limited nucleation sites and slow crystal growth within the unfilled PLA/PHA matrix [41,42]. Furthermore, the low crystallinity reflects the inherently restricted crystallization ability of PLA due to its rigid molecular structure and relatively slow crystallization kinetics. The broad cold crystallization peak observed for S0 is characteristic of incomplete crystallization during cooling, which subsequently occurs upon reheating. The incorporation of MCC significantly altered the crystallization behavior of the composites. For samples S1–S4 containing increasing MCC contents without compatibilizer, the Tgshowed a slight decrease from 60.1 °C to 58.9 °C, while Tcc gradually decreased from 115.2 °C to 111.4 °C. This reduction in Tcc denotes that MCC particles acted as heterogeneous nucleating agents, promoting crystal formation at lower temperatures. The melting temperature (Tm) of the PLA/PHA/MCC biocompositesremained nearly constant for all formulations, ranging from 168.4 to 169.9 °C indicating that the incorporation of MCC and the compatibilizer did not significantly alter the crystal structure of the polymer matrix. A slight increase in Tm was observed for S10 (169.6 °C) compared with the neat PLA/PHA blend (168.4 °C), which is attributed to improved interfacial adhesion and enhanced crystalline stability resulting from the combined presence of MCC and the compatibilizer. The stronger matrix–filler interactions likely promote the formation of more thermally stable crystallites that require slightly higher temperatures to melt. Since the observed increase is only 1.2 °C, it reflects a modest improvement in crystalline stability rather than a significant change in the crystal structure. The presence of MCC provides numerous nucleationsites that promote the ordered arrangement of polymer chains, thereby accelerating crystallization. Consequently, the crystallinity increased progressively from 28.6% for S0 to 38.3% for S4, representing an enhancement of approximately 34% compared with the neat blend. Similarly, the melting enthalpy (ΔHm) increased from 29.8 J g^−1^ to 36.8 J g^−1^, confirming the formation of a greater proportion of crystalline regions [43,44]. The melting temperature also exhibited a slight increase from 168.4 °C to 169.4 °C, suggesting improved crystal perfection and stability as a result of MCC-induced nucleation.

The influence of the compatibilizer is clearly evident in samples S5–S8. Compared with the corresponding non-compatibilized formulations, these composites exhibited lower Tcc values and higher crystallinity. The compatibilizer promotes interfacial adhesion by improving compatibility between hydrophilic MCC particles and the hydrophobic PLA/PHA matrix thereby facilitating uniform filler distribution and more efficient transfer of applied stresses. Better dispersion increases the effective surface area of MCC available for nucleation, thereby promoting crystal growth throughout the matrix. Also, The cold crystallization enthalpy (ΔHcc) gradually decreased from 15.0 J g^−1^ for the neat PLA/PHA blend (S0) to 11.4 J g^−1^ for the S7 composite indicating that the incorporation of MCC together with the compatibilizer promoted more effective crystallization during processing. Among all formulations, S7 (6 wt.% MCC and 2 wt.% malaeic anhydride compatibilizer) exhibited the most favorable thermal characteristics. This sample showed the lowest cold crystallization temperature (110.5 °C), highest melting temperature (170.0 °C), highest melting enthalpy (38.9 J g^−1^) and maximum crystallinity (40.1%). The reduction in Tcc by approximately 4.7 °C relative to S0 indicates a substantially enhanced crystallization rate, while the increase in crystallinity by approximately 11.5% demonstrates the effectiveness of MCC and compatibilizer in promoting crystal formation. The sharper and more intense melting peak observed for S7 further suggests the development of a more uniform and well-organized crystalline structure. When the malaeic anhydride compatibilizer content was increased further in S9 to S11 specimen, the crystallinity remained higher than that of the neat blend but did not exceed that of S7. The crystallinity values ranged from 33.1% to 38.9%, while Tcc remained between 110.8 °C and 112.5 °C. Although higher compatibilizer concentrations continued to improve matrix–filler interactions, excessive compatibilizer may have partially restricted polymer chain mobility or interfered with crystal packing, thereby limiting further crystalline development. Similarly, increasing MCC content beyond the optimum level may promote particle agglomeration, reducing the availability of effective nucleation sites and hindering uniform crystal growth. As a result, S8 and S11 showed slightly lower crystallinity values (38.7% and 38.9%, respectively) compared with S7 (40.1%). The DSC results correlate well with the structural observations obtained from XRD analysis. The increase in crystallinity measured by DSC closely follows the crystallinity index determined from XRD, confirming that MCC promotes crystal nucleation and growth within the PLA/PHA matrix [44].

**Figure 5:**
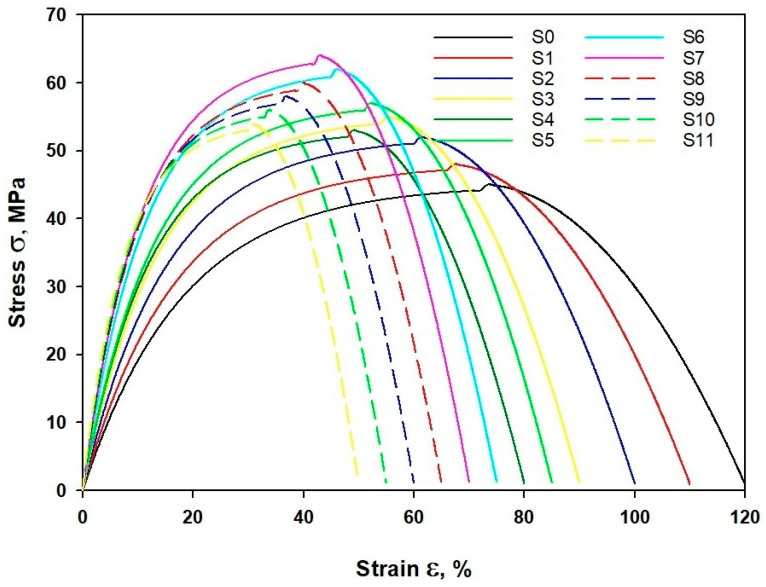
Representative tensile stress–strain curves of PLA/PHA/MCC biocomposites.

The representative tensile stress–strain curves of all formulations are demonstrated in Figure 5, whereas the extracted mechanical characteristics are displayed in Table 4.

The neat PLA/PHA blend (S0) exhibited a tensile strength of 45 ± 0.82 MPa, Young’s modulus of 1500 ± 2.80 MPa, elongation at break of 120 ± 2.0%, and impact strength of 3.5 ± 0.21 kJ/m^−2^. The relatively low tensile strength and modulus indicate the limited load-bearing capability of the unreinforced matrix, while the high elongation at break reflects the ductile nature imparted by the PHA component. With the addition of MCC from 2 wt.% (S1) to 6 wt.% (S3), the tensile strength increased progressively from 48 ± 0.74 MPa to 55 ± 0.76 MPa, while the Young’s modulus increased from 1650 ± 2.91 MPa to 2000 ± 2.56 MPa. Simultaneously, elongation at break decreased from 110 ± 1.7% to 90 ± 2.3%, indicating that the rigid MCC particles restricted polymer chain mobility. The impact strength also improved from 3.8 ± 0.24 to 4.6 ± 0.41 kJ/m^2^ suggesting effective stress dissipation through well-dispersed cellulose particles. These improvements can be credited to the nucleating effect of MCC and enhanced interfacial stress transfer between the cellulose reinforcement and the PLA/PHA matrix [45]. At 8 wt.% MCC (S4), the tensile strength slightly decreased to 53 ± 0.81 MPa, despite the modulus increasing further to 2100 ± 1.98 MPa. The reduction in strength and impact resistance of 4.4 ± 0.32 kJ/m^2^ indicates the onset of MCC agglomeration, which creates stress concentration sites and limits the efficiency of load transfer. This observation is consistent with SEM micrographs showing localized particle clustering at higher MCC loading. The introduction of a compatibilizer appreciably enhance the mechanical performance of the composites. Sample S5 exhibited a tensile strength of 57 ± 0.89 MPa, modulus of 2200 ± 1.89 MPa, and impact strength of 4.8 ± 0.43 kJ/m^2^ are higher than those of the corresponding uncompatibilized formulation. Similar improvements were observed for S6, where tensile strength increased to 62 ± 0.87 MPa, modulus to 2400 ± 1.99 MPa, and impact strength to 5.5 ± 0.41 kJ/m^2^. These enhancements indicate improved interfacial adhesion between MCC and the PLA/PHA matrix, resulting in more efficient stress transfer and reduced particle pull-out during deformation. Among all specimens, S7 exhibited the optimum mechanical performance, achieving the highest tensile strength of 64 ± 0.79 MPa and impact strength of 5.8 ± 0.34 kJ/m^2^ together with a high Young’s modulus (2500 ± 2.43 MPa). Compared with neat PLA/PHA (S0), S7 demonstrated approximately 42.2% improvement in tensile strength, 66.7% increase in stiffness, and 65.7% enhancement in impact resistance. The superior performance of S7 can be credited to the synergistic effect of the optimal MCC concentration and compatibilizer-assisted dispersion, which promotes strong matrix–filler interactions and efficient stress transfer. The excellent mechanical performance of S7 is further supported by XRD results showing the highest crystallinity index (40.1%) and DSC analysis indicating enhanced crystallization behavior. For S8 (8 wt.% MCC + malaeic anhydride compatibilizer), the tensile strength decreased slightly to 60 ± 0.91 MPa, although the modulus increased to 2600 ± 2.35 MPa. The reduction in strength and impact resistance 5.2 ± 0.29 kJ/m^2^ suggests that excessive MCC loading promotes particle aggregation, which offsets the reinforcing effect despite the presence of compatibilizer. The high-compatibilizer formulations (S9–S11) showed a different trend. The modulus continued to increase from 2700 ± 1.98 MPa (S9) to 2900 ± 2.87 MPa (S11), indicating increased rigidity of the composite structure. However, tensile strength gradually decreased from 58 ± 0.69 MPa to 54 ± 0.85 MPa, while elongation at break declined from 60% to 50% and impact strength from 4.9 ± 0.18 kJ/m^2^ to 4.3 ± 0.45 kJ/m^2^. The reduction in strength and toughness despite higher stiffness suggests that excessive compatibilizer content may lead to increased matrix rigidity and reduced molecular mobility, resulting in a more brittle material response [46]. The mechanical results reveal a clear balance between stiffness and toughness in the PLA/PHA/MCC system. MCC effectively enhances tensile strength and modulus through reinforcement and nucleation effects, while the compatibilizer improves filler dispersion and interfacial adhesion. The optimum formulation was identified as S7 (6 wt.% MCC + compatibilizer), which exhibited the best combination of strength (64 MPa), stiffness (2500 MPa), toughness (5.8 kJ/m^2^, and crystallinity (40.1%).

**Figure 6:**
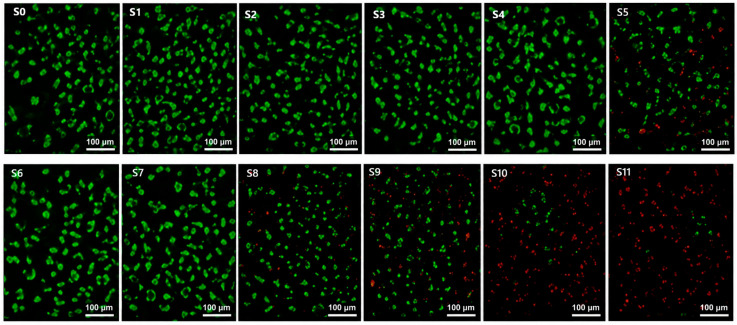
Fluorescence microscopy images of live/dead cell staining on PLA/PHA/MCC biocomposites.

Figure 6 presents fluorescence microscopy images of PLA/PHA/MCC biocomposites after biological exposure, where green fluorescence represents viable microbial cells (live cells) and red fluorescence indicates non-viable or damaged microbial cells (dead cells). The neat PLA/PHA blend (S0) exhibited predominantly green fluorescent cells distributed across the specimen surface, indicating a high population of viable microorganisms with limited cellular damage. Similar behavior was observed for S1–S4 where the incorporation of MCC promoted microbial attachment and colonization due to the hydrophilic nature of cellulose. The increased availability of hydroxyl groups on the MCC surface enhanced water absorption and surface wettability creating favorable conditions for microbial growth [44]. Consequently, the density of green fluorescent cells gradually increased from S1 to S4, suggesting that moderate MCC incorporation promotes microbial adhesion and biofilm formation. The higher microbial population observed in S3 and S4 indicates enhanced biodegradation potential compared with the neat polymer matrix. A distinct change in microbial behavior was observed for the compatibilized systems (S5–S8). While green fluorescence remained dominant in S5 and S6, the appearance of scattered red fluorescent cells indicates the initiation of cellular stress and membrane damage. The improved dispersion of MCC and stronger matrix–filler interactions resulting from compatibilizer addition created a more homogeneous surface morphology, facilitating uniform microbial colonization [47]. Among these specimens, S7 exhibited the highest density of uniformly distributed viable cells with minimal clustering, indicating an optimal balance between microbial attachment and material integrity. This observation is consistent with the superior interfacial adhesion, crystallinity, and thermal stability previously identified through SEM, FTIR, XRD, DSC, and TGA analyses. The homogeneous distribution of viable microorganisms suggests that S7 provides the most favorable environment for controlled biodegradation.

For S8, a noticeable increase in red fluorescence was observed compared with S7. This behavior is attributed to the higher MCC concentration, which may have led to localized filler aggregation and heterogeneous degradation zones. Such regions can alter nutrient diffusion and microbial accessibility, resulting in increased cellular stress and partial loss of viability. Nevertheless, the coexistence of green and red cells confirms active microbial interaction with the composite surface. The specimens containing higher compatibilizer concentrations (S9–S11) exhibited a substantial increase in red fluorescent cells accompanied by a reduction in green fluorescence. In S9, both viable and non-viable cells were present, indicating an intermediate stage of microbial activity. However, S10 and particularly S11 showed predominantly red fluorescence, suggesting extensive cell membrane disruption and reduced microbial viability. The increased proportion of dead cells may be associated with advanced biodegradation processes, accumulation of degradation by-products, local environmental changes near the composite surface, or altered nutrient availability resulting from enhanced polymer degradation [46,47,48,49,50]. The reduction in viable cell density indicates that the surface conditions become less favorable for sustained microbial proliferation at excessive compatibilizer and MCC loadings. The fluorescence microscopy results demonstrate that MCC incorporation significantly influences microbial attachment and biodegradation behavior of PLA/PHA composites. Moderate MCC contents combined with compatibilizer addition promote uniform microbial colonization and enhanced biodegradation activity, whereas excessive MCC and compatibilizer concentrations increase cellular stress and reduce microbial viability. Among all formulations, S7 (7 wt.% MCC with compatibilizer) exhibited the most balanced microbial response, characterized by a high density of viable cells, uniform distribution, and limited cell death. These findings correlate strongly with the structural, thermal, and morphological analyses, confirming that S7 provides the optimum combination of filler dispersion, interfacial compatibility, crystallinity, and biodegradation performance.

**Figure 7:**
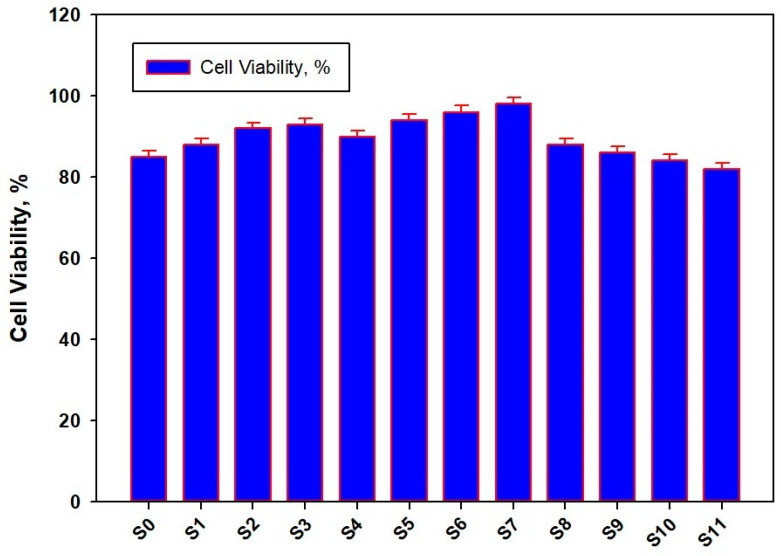
Cell viability evaluation of PLA/PHA/MCC biocomposites.

The cytocompatibility of the PLA/PHA/MCC biocomposites was evaluated using cell viability as presented in Figure 7. All formulations exhibited cell viability values above 80%, indicating good biocompatibility and absence of severe cytotoxic effects according to ISO 10993 standards [51], where materials with cell viability exceeding 70% are generally considered non-cytotoxic [52,53,54,55].

The neat PLA/PHA blend (S0) exhibited a cell viability of approximately 85 ± 1.45%, which increased progressively with MCC incorporation. Samples S1, S2, and S3 showed viability values of approximately 88 ± 1.45%, 91 ± 1.41%, and 92 ± 1.37%, respectively, suggesting that low-to-moderate MCC loading promotes favorable cell attachment and proliferation. The presence of hydroxyl rich MCC particles likely enhanced surface hydrophilicity and protein adsorption, creating a more favorable environment for cell growth [56,57]. A slight decrease was observed for S4 (90 ± 1.42%), possibly due to the onset of particle agglomeration at higher MCC loading. The compatibilized systems (S5–S8) demonstrated further improvement in cell viability, reaching 93 ± 1.66%, 95 ± 1.58%, and 98 ± 1.55% for S5, S6, and S7, respectively. Among all formulations S7 (6 wt.% MCC with compatibilizer) exhibited the highest cell viability, indicating optimal filler dispersion and superior matrix–filler interactions. The enhanced cellular response observed for S7 is consistent with the SEM, DSC, and XRD results, which demonstrated improved filler distribution, higher crystallinity, and stronger interfacial adhesion. However, further increasing MCC concentration to 8 wt.% (S8) resulted in a slight reduction in viability to approximately 88%, suggesting that excessive filler content may adversely affect surface homogeneity [46,48]. Similarly, the high-compatibilizer formulations from S9 to S11 exhibited gradual decreases in viability from 86 ± 1.61% to 82 ± 1.55%, indicating that excessive compatibilizer content may alter surface chemistry and reduce cellular proliferation.

**Figure 8:**
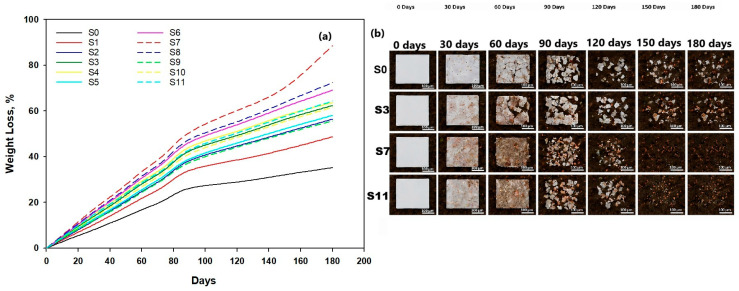
Soil burial biodegradation behavior of PLA/PHA/MCC biocomposites. (**a**) Weight loss (%) of formulations S0–S11 as a function of soil burial time (0–180 days). (**b**) Representative optical images of selected specimens (S0, S3, S7, and S12).

Figure 8a,b presents the soil burial biodegradation performance of PLA/PHA/MCC biocompositesevaluated over 180 days through weight-loss measurements and visual observation of specimen degradation. The soil-burial test provides an indication of the degradation behavior of the developed biocomposites under natural environmental conditions. However, the obtained weight loss should be interpreted as evidence of material disintegration and degradation rather than complete biodegradation, since mineralization, CO_2_ evolution, and standardized biodegradation measurements were not performed in this study. The results clearly demonstrate that all formulations undergo progressive biodegradation with increasing burial time; however, the degradation rate strongly depends on MCC content and compatibilizer concentration. The neat PLA/PHA blend (S0) exhibited the lowest degradation rate, reaching only 35.2% weight loss after 180 days. The gradual increase in weight loss from 4.2% at 15 days to 35.2% at 180 days indicates the inherently slow degradation of the polymer matrix due to its relatively hydrophobic nature and compact structure. Incorporation of MCC significantly accelerated biodegradation. For specimens without compatibilizer, weight loss increased from 48.7% (S1, 2% MCC) to 56.4% (S2, 4% MCC), 62.4% (S3, 6% MCC) and 63.7% (S4, 8% MCC) after 180 days. The enhanced degradation can be attributed to the hydrophilic cellulose particles, which facilitate moisture absorption, microbial colonization, and hydrolytic cleavage of ester bonds within the PLA/PHA matrix [58]. The addition of 2 wt.% malaeic anhydride compatibilizer further promoted biodegradation. Specimens S5, S6, S7, and S8 exhibited weight losses of 58.1%, 69.1%, 88.5%, and 72.4%, respectively, after 180 days. Among all formulations, S7 (6 wt.% MCC + 2 wt.% compatibilizer) displayed the highest biodegradation rate, achieving 88.5% weight loss, which is approximately 2.5 times higher than that of neat PLA/PHA. The degradation profile of S7 increased steadily from 8.6% (15 days) to 17.2% (30 days), 25.0% (45 days), 33.5% (60 days), 41.5% (75 days), 50.6% (90 days), 60.2% (120 days), 70.2% (150 days), and finally 88.5% (180 days). This superior biodegradation behavior suggests that the optimized combination of MCC and compatibilizer generated a more homogeneous microstructure with enhanced water penetration pathways and increased accessibility to microorganisms. For composites containing 4 wt.% malaeic anhydride compatibilizer, biodegradation remained higher than neat PLA/PHA but lower than the optimum S7 formulation. The final weight losses were 55.6% (S9), 61.6% (S10), and 64.3% (S11) after 180 days. The slight reduction in degradation compared with S7 indicates that excessive compatibilizer may improve interfacial adhesion and structural compactness, thereby limiting water diffusion and microbial attack.

The optical micrographs of selected formulations, Figure 8b, provide direct evidence of progressive biodegradation during soil burial. At 0 days, all specimens possessed smooth, intact, and homogeneous surfaces with no visible defects. After 30 days, slight discoloration and surface roughening became apparent, indicating the initiation of moisture uptake and microbial activity. By 60 days, visible cracks, erosion marks, and fragmentation appeared, particularly in the MCC-containing composites. The degradation became much more pronounced after 90 days, where substantial fragmentation of the specimens was observed. The extent of fragmentation was significantly greater for S7 compared with the other formulations, indicating accelerated structural deterioration. After 120 days, most specimens showed extensive disintegration into smaller fragments, while S7 exhibited severe breakdown with only scattered residual pieces remaining. At 180 days, S7 was almost completely decomposed, leaving only trace residues within the soil matrix, whereas S0 still retained recognizable fragments, confirming its slower degradation rate.

The soil burial results correlate well with the FTIR, XRD, DSC, and SEM analyses. The higher crystallinity and improved filler dispersion observed for S7 created a balanced microstructure that enhanced both mechanical performance and biodegradation behavior. The hydrophilic nature of MCC increased water absorption, while the compatibilizer improved interfacial interactions, promoting uniform degradation throughout the matrix [57,58]. Consequently, S7 achieved the optimum combination of structural integrity during service and rapid biodegradation after disposal.

## 4. Conclusions

This study successfully developed and characterized a series of sustainable PLA/PHA/MCC biocomposites containing varying MCC loadings and compatibilizer concentrations (S0–S11). The results demonstrated that the integration of microcrystalline cellulose (MCC) significantly influenced the structural, thermal, mechanical, and biological performance of the PLA/PHA matrix. FTIR analysis confirmed the occurrence of robust intermolecular interactions between the polymer matrix and MCC through hydrogen bonding, while XRD results revealed enhanced crystalline organization with increasing MCC content. The crystallinity index increased from 28.6% for S0 to a maximum of 40.1% for S7, indicating the effective nucleating role of MCC and the beneficial effect of compatibilizer on crystal growth. Thermal characterization by TGA/DTG and DSC further confirmed the reinforcing effect of MCC. The thermal degradation temperature increased from 325.1 °C (S0) to 343.8 °C (S7), accompanied by a substantial increase in char residue from 5.6% to 16.8%, indicating enhanced thermal stability and improved resistance to thermal decomposition. DSC results showed increased crystallization efficiency and crystal perfection with MCC addition, where S7 exhibited the most favorable crystallization behavior, consistent with the highest crystallinity observed in XRD analysis. However, excessive MCC loading (8 wt.%) caused a slight reduction in thermal and crystalline properties due to filler agglomeration and restricted polymer-chain mobility. Morphological observations from SEM demonstrated that MCC particles were uniformly distributed within the PLA/PHA matrix at moderate filler contents, particularly for compatibilized formulations. S7 displayed the most homogeneous particle dispersion and strongest matrix–filler interaction, whereas higher MCC concentrations (S8 and S11) resulted in visible particle agglomeration. Cell attachment studies further confirmed excellent biocompatibility of the developed biocomposites, with S6 and S7 supporting extensive cell spreading, adhesion, and proliferation. At higher MCC concentrations, cell viability decreased suggesting that particle agglomeration negatively affected the biological response. Mechanical testing revealed a significant enhancement in strength and stiffness with MCC incorporation. Tensile strength increased from 45 MPa (S0) to 64 MPa (S7), while Young’s modulus increased from 1500 MPa to 2500 MPa. Impact strength also improved from 3.5 to 5.8 kJ/m^2^, confirming efficient stress transfer between MCC and the polymer matrix. Although elongation at break decreased from 120% to 70%, the resulting composites exhibited a favorable balance between stiffness and toughness. Beyond the optimum MCC concentration, mechanical performance slightly deteriorated due to particle aggregation and reduced interfacial efficiency. Overall, the combined results demonstrate that the synergistic incorporation of 6 wt.% MCC and compatibilizer (S7) provides the best overall performance, exhibiting the highest crystallinity, superior thermal stability, enhanced mechanical properties, excellent filler dispersion, and outstanding biocompatibility. These findings confirm that MCC is an effective sustainable bio-reinforcement for PLA/PHA blends and highlight the potential of the developed biocomposites for advanced biomedical, packaging, tissue engineering, and environmentally sustainable engineering applications requiring high mechanical performance, thermal stability, and biological safety.

## Figures and Tables

**Table 1:** Composition of PLA/PHA/MCC biocomposite formulations (wt.%).

Specimen	PLA, wt.%	PHA, wt.%	MCC, wt.%	Compatibilizer, wt.%
S0	70	30	0	0
S1	68	30	2	0
S2	66	30	4	0
S3	64	30	6	0
S4	62	30	8	0
S5	66	30	2	2
S6	64	30	4	2
S7	62	30	6	2
S8	60	30	8	2
S9	62	30	4	4
S10	60	30	6	4
S11	58	30	8	4

**Table 2:** Characteristic thermal degradation parameters of the PLA/PHA/MCC biocomposites.

Specimen	Tonset (°C)	Tmax (°C)	Residue at 800 °C (%)
S0	301.2 ± 0.25	325.1 ± 0.23	5.6 ± 0.31
S1	306.8 ± 0.18	328.7 ± 0.21	7.1 ± 0.29
S2	310.4 ± 0.15	331.2 ± 0.24	8.7 ± 0.23
S3	315.6 ± 0.16	334.5 ± 0.23	10.1 ± 0.15
S4	319.2 ± 0.21	337.6 ± 0.20	11.8 ± 0.27
S5	309.7 ± 0.14	332.8 ± 0.27	13.4 ± 0.18
S6	318.2 ± 0.13	340.2 ± 0.30	15.1 ± 0.29
S7	323.6 ± 0.19	343.8 ± 0.21	16.8 ± 0.31
S8	320.4 ± 0.16	339.1 ± 0.2	19.0 ± 0.25
S9	311.2 ± 0.23	333.6 ± 0.23	21.3 ± 0.24
S10	315.0 ± 0.16	336.1 ± 0.25	24.6 ± 0.19
S11	317.8 ± 0.17	336.8 ± 0.29	24.9 ± 0.14

**Table 3:** Thermal characteristics of PLA/PHA/MCC biocomposites.

Specimen	Tg, °C	Tm, °C	Tcc, °C	ΔHm, J/g	ΔHcc, J/g	Xc, %
S0	60.1	168.4	115.2	29.8	15.0	28.6
S1	59.8	168.7	114.3	31.5	14.8	31.2
S2	59.5	169.0	113.2	33.4	13.9	33.8
S3	59.2	169.2	112.0	35.6	13.0	36.9
S4	58.9	169.4	111.4	36.8	12.2	38.3
S5	59.4	169.1	113.0	32.8	13.6	32.6
S6	59.0	169.5	111.8	35.2	12.3	36.6
S7	58.6	170.0	110.3	38.9	11.4	40.1
S8	58.5	169.8	110.9	37.4	11.9	38.7
S9	59.3	169.2	112.5	33.0	13.8	33.1
S10	58.9	169.6	111.6	35.8	12.9	37.2
S11	58.4	169.9	110.8	37.8	11.8	38.9

**Table 4:** Mechanical characteristics of PLA/PHA/MCC.

Specimen	Tensile Strength, MPa	Young’s Modulus, MPa	Elongation at Break, %	Impact Strength, KJ/m^2^
S0	45 ± 0.82	1500 ± 2.80	120 ± 2.0	3.5 ± 0.21
S1	48 ± 0.74	1650 ± 2.91	110 ± 1.7	3.8 ± 0.24
S2	52 ± 0.69	1800 ± 3.42	100 ± 1.9	4.2 ± 0.36
S3	55 ± 0.76	2000 ± 2.56	90 ± 2.3	4.6 ± 0.41
S4	53 ± 0.81	2100 ± 1.98	80 ± 1.8	4.4 ± 0.32
S5	57 ± 0.89	2200 ± 1.89	85 ± 1.5	4.8 ± 0.43
S6	62 ± 0.87	2400 ± 1.99	75 ± 2.3	5.5 ± 0.41
S7	64 ± 0.79	2500 ± 2.43	70 ± 1.9	5.8 ± 0.34
S8	60 ± 0.91	2600 ± 2.35	65 ± 1.4	5.2 ± 0.29
S9	58 ± 0.69	2700 ± 1.98	60 ± 1.4	4.9 ± 0.18
S10	56 ± 0.78	2800 ± 2.45	55 ± 1.7	4.6 ± 0.37
S11	54 ± 0.85	2900 ± 2.87	50 ± 1.9	4.3 ± 0.45

## Data Availability

The original contributions presented in this study are included in the article. Further inquiries can be directed to the corresponding author.
